# Covid 19, gender violence, depression, post-traumatic stress disorder, posttraumatic stress disorder

**DOI:** 10.1192/j.eurpsy.2022.2271

**Published:** 2022-09-01

**Authors:** J. Castillo, M. Llamuca, V. Valdéz

**Affiliations:** 1 Universidad Católica de Santiago de Guayaquil, Facultad De Ciencias Médicas - Carrera De Medicina, Guayaquil, Ecuador; 2 Universidad Católica de Santiago de Guayaquil, Ciencias De La Salud - Facultad De Medicina, Guayaquil, Ecuador

**Keywords:** PTSD, COVID19, Depression, SARS-CoV-2

## Abstract

**Introduction:**

In Ecuador, the first case of covid19 was reported on February 29^th^ of 2020, forcing people to remain in lockdown, which increased gender violence; post-traumatic stress disorder (PTSD) and depression.

**Objectives:**

Determine the cases during the lockdown caused by the covid-19 pandemic we found victims of some type of gender-based violence, depression, and PTSD.

**Methods:**

An observational, cross-sectional descriptive study was carried out based on surveys conducted online, the study was conducted on January 29^th^ of 2021, in the province of Guayas-Ecuador. Performed with “google forms” platform, data on affiliation, the situation of intimate partner violence and the Davidson trauma test (PTSD) and the Beck test (depression) were collected.

**Results:**

A total of 411 samples were obtained, classified according to age, sex, number of children, education, occupation, intimate partner relationship, whether they had suffered gender violence and types of violence. 88 (21.41%) people reported having suffered some type of violence, of which 25 (28.42%) were men and 62 (70.45%) were women. The most common was psychological with 53 (60.23%) people. 82 respondents tested positive to Davidson test for PTSD, equivalent to 20% of the total sample. 51 people (12%) reported suffering from mild depression, 53 (13%) reported suffering from moderate depression and 38 people (9%) reported major depression with Beck test.

**Conclusions:**

In this study we evidenced that although the interviewers claimed they had never suffered gender violence , it was observed that the results were incongruent, so it is important to highlight that talking about gender violence is still considered a stigma in our society.

**Disclosure:**

No significant relationships.

